# Association between problematic social media use and memory performance in a sample of Lebanese adults: the mediating effect of anxiety, depression, stress and insomnia

**DOI:** 10.1186/s13005-021-00260-8

**Published:** 2021-02-23

**Authors:** Maya Dagher, Youssef Farchakh, Sam Barbar, Chadia Haddad, Marwan Akel, Souheil Hallit, Sahar Obeid

**Affiliations:** 1 Faculty of Medicine and Medical Sciences, Holy Spirit University of Kaslik (USEK), Jounieh, Lebanon; 2 Univ. Limoges, UMR 1094, Neuroépidémiologie Tropicale, Institut d’Epidémiologie et de Neurologie Tropicale, GEIST, 87000 Limoges, France; 3 Research and Psychology Departments, Psychiatric Hospital of the Cross, Jal Eddib, Lebanon; 4 INSPECT-LB: Institut National de Sante Publique, Epidemiologie Clinique et Toxicologie-Liban, Building 560, Street 8, Biakout, Beirut, Lebanon; 5 School of Pharmacy, Lebanese International University, Beirut, Lebanon; 6 Faculty of Arts and Sciences, Holy Spirit University of Kaslik (USEK), Jounieh, Lebanon

**Keywords:** Problematic social media use, Memory performance, Depression, Anxiety, Stress, Insomnia

## Abstract

**Background:**

Psychological factors such as depression, anxiety, stress and insomnia and problematic social media use are able to alter our memories and might have an impact on memory function and retrieval. More studies are needed to better understand the relationship between memory performance and mental health disorders, especially the ones that could be related to problematic social media use. The objective of this study was to evaluate any association between problematic social media use, depression, anxiety, stress, and insomnia vs memory performance among a representative sample of Lebanese people.

**Methods:**

This cross-sectional study, conducted between January and May 2019, enrolled 466 community dwelling participants using a proportionate random sample from all Lebanese governorates. The questionnaire consisted of the following measures: the Memory Awareness Rating Scale (MARS) to assesses views of memory performance, the problematic social media use scale to measure the degree of addiction to social media, the Hamilton depression rating scale and Hamilton anxiety scale to assess depression and anxiety respectively, the Beirut Distress Scale to assess stress and the Lebanese Insomnia sale to assess insomnia. The data analysis was performed using the SPSS software version 25. A linear regression was conducted, taking the memory performance scale as the dependent variable. A mediation analysis was performed to test the effect of problematic social media use on memory performance mediated by depression, anxiety, stress and insomnia.

**Results:**

Higher problematic social media use (Beta = − 0.21) and higher anxiety (Beta = − 0.25) were significantly associated with lower memory performance. The association between problematic social media use and memory performance was partially mediated by anxiety (21.19%) but not depression, stress or insomnia.

**Conclusion:**

Concerning problematic social media use, a clear correlation was demonstrated in this study linking it to lower memory performances. Future studies should evaluate the possible mechanisms and methods for effective awareness especially towards the younger generation.

**Supplementary Information:**

The online version contains supplementary material available at 10.1186/s13005-021-00260-8.

## Background

Considered as one of the most essential aspects of life, memory is the brain’s filling system. It contains everything a person has learned in the past [1]. Memory could be best defined as the mental capacity to retain and preserve information, in order to retrieve them when needed. To go more in depth, memory consists of three main types, which cooperate together in forming lasting information [2]. Our sensory memory allows to retain sensory data after the stimulus has disappeared, such as smells and voices. Our short-term memory holds information only for a few seconds, and our long-term memory allows us to store information for long lapses of time, sometimes up to a lifetime. Researchers have always given serious attention to the factors that influence memory processing in humans [3] and these factors can also be split into two big categories: organic illnesses such as tumors, molecular abnormalities, neuronal damage, scarcity and over activity, and psyche related disorders such as mental and emotion-related conditions. New studies suggest that a variety of mental health disorders, along with daily habits and behaviors, come into play when considering memory performance and forgetfulness [4]. That being said, given how most mental health conditions are characterized by both organic and psychological underlying abnormalities we couldn’t help but ask, could certain mental health disorders affect our memory performance?

In recent years, social media use has remarkably increased and has reached over 2 billion users worldwide [5, 6]. De facto, according to a study made in 2018, 45% of adolescents and young adults declared being constantly online [5, 6]. Despite the massive benefits of technology such as fast access to information, easy connection with communities all over the world, entertainment and business development, increasing use of social network sites can lead to the potential risk of addiction, known as “problematic social media use”. Over and above that, a study targeting adolescent social media use pinpointed the different impacts of active and passive social media use on teenagers [7]. The influence of pathological social media use on cognitive functions and memory remains a central topic of investigation. For example, a study conducted by Liu et al. confirmed that reliance on online searching and excessive social media use impede memory retrieval by reducing the functional connectivity and synchronization of associated brain regions [8].

Moreover, depression, anxiety, stress and insomnia are common disorders in primary care settings, with high chances of co-existence. According to a study conducted among American adults in 2006, the prevalence of depression increased from 3.33 to 7.06% in a period of 10 years [9]. Decades of research have investigated the impact of depression on memory. In fact, Schweizer et al. linked symptoms of depression with subjective memory complaints [10]. Additionally, results of a study conducted by Balderston et al. suggest that cognitive and memory deficits may represent a key component of clinical anxiety [11]. Additionally, a systematic review showed that all four studied domains of social media (Time spent, activity, investment, and addiction) were correlated with depression, anxiety, and psychological disturbances [12]. Furthermore, a recent and growing body of evidence has indicated that sleep is related to cognitive abilities [13]. For instance, Cohen-Zion et al. proved that among patients with chronic insomnia, reductions in neurocognitive performance are associated with increasingly severe sleep disorders over time [14]. Last but not least, studies have shown that adrenal hormones such as catecholamine and glucocorticoids are released into the organism in response to stress. These hormones affect the limbic system in the brain and thus can affect our memory function in different ways. It has been shown that endogenous catecholamine secretion strengthen memory in contrast to glucocorticoids. The latter was the topic of many controversial findings; some suggests that these hormones enhance memory performance others advocate their potential disruptive effect [15], making the search for the truth ever so important.

**Fig. 1:**
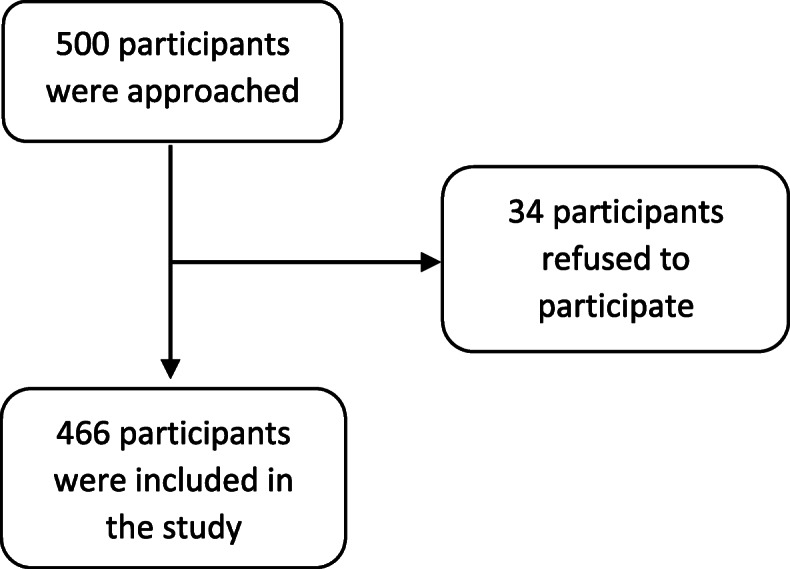
Flow chart of the participants included in the study

Aiming to better understand the phenomena at play, a theoretical framework was deducted based on extensive literature research and illustrated in Fig. 1 below. First, social networking, media consumption and naturally therefore Problematic Social Media Use (PSMU), were positively correlated with lower memory performance. These findings applied to both quality/clarity of memory recollection as well as quantity of memories stored [16–18]. Second, emotional disorders such as depression, insomnia and stress were also shown to have deleterious consequences on our memory function [19, 20]. That being said, the effects of stress are regulated by numerous significant factors and thus it is of importance to note that according to recent studies, stress adversely affects some episodic memory processes whilst improving others [21]. Concerning anxiety, controversial results were elucidated: some studies emphasis that anxiety did not induce lower memory performance (in contrast to depression), whilst others attempt to explain how anxiety has the same repercussions on memory as the other mood disorders [22]. Finally, people suffering from these psychiatric disorders, more specifically emotional disorders, are more prone to develop addictive disorders such as PSMU [23]. To boot, previous results indicated that social anxiety lead to favoring computer mediated over face to face communication [24]. Reciprocally, individuals with greater PSMU levels neglect some aspects of their lives thus contributing to the development of depressive symptoms. With all that in mind, given how the literature illustrates this complex relationship suggests that certain mental health disorders like anxiety, depression, stress and insomnia might play an intermediate role between PSMU and memory performance, hence mediating their correlation.

To summarize, memory is fundamentally a vital biological function crucial for our survival. The progress that has been made in the past few decades elucidating the factors affecting memory and forgetfulness has provided useful insight into how memories might be enhanced [25]. Nevertheless, more studies are needed to better understand the relationship between memory performance and certain mental health disorders, particularly depression, anxiety, stress and insomnia. What’s more, considering our society’s newly found interest in social networking and given the progressive unraveling of its adverse effects on our psyche and cognitive skills [26], we found great potential in analyzing the relationship between memory function and Problematic Social Media Use (PSMU). In addition, research projects taking on similar subjects in Lebanon are still scarce, hence underlining the necessity of diving deeper into this matter and making the pursuit of this topic ever so compelling. Finally, bearing in mind that mental disorders are among the most common health problems in Lebanon [27], it is of great importance to highlight and expose their potential repercussions, notably on our memory. For all of that, the objective of this study was to evaluate any association between problematic social media use, depression, anxiety, stress, and insomnia vs memory performance among a representative sample of Lebanese people.

## Methods

### Study design and participants

This cross-sectional study was carried out between January and May 2019. It enrolled 466 residents of the community randomly selected from Lebanon’s Mohafazat in a proportionate rate. The Mohafazat are divided into Caza (stratum), divided into villages. From a list provided by the Central Agency of Statistics in Lebanon, we chose two villages per Caza where the questionnaire was distributed randomly to the households, based on a random sampling technique to select the included house. Those who agreed to take part in the study were invited to complete the questionnaire via a face-to-face interview. All individuals over the age of 18 were eligible to participate. Excluded were those with dementia (according to one of the family members), and those who refused to complete the questionnaire. The distribution of the questionnaire as well the data collection were performed by a one study-independent well-trained clinical psychologist. The clinical psychologist has gone through a training with the researcher before starting data collection. Any queries or doubts that were encountered during the data collection, the psychologist had the capacity to contact and refer to the researcher. Paper-based questionnaire were distributed to the participants. The required time to complete the questionnaire was around 10–15 min. The methodology used in this study is similar to the one used in previous papers [28–31].

Out of 500 questionnaires distributed, 466 (93.2%) were completed and collected back. The sociodemographic characteristics of the participants are summarized in Table 1. The results showed that the mean age of the participants was 27.29 ± 11.46 years and the mean number of hours spent on social media per day was 6.22 ± 4.92. The majority of the participants were females (61.8%), had a university level of education (66.7%), single (68.1%), with a low monthly income (61.4%). Almost all participants use their cellular as the mostly used device on social media (92.9%) and 19.4% were smokers.

**Table 1:** Sociodemographic characteristics of the sample population (*N* = 466)

	**Frequency**	**Percentage**
**Gender**		
Male	176	38.2%
Female	285	61.8%
**Education level**		
Illiterate	8	1.8%
Primary	17	3.7%
Complementary	32	7.0%
Secondary	95	20.8%
University	304	66.7%
**Monthly income**		
< 1000 $	266	61.4%
1000–2000 $	122	28.2%
> 2000 $	45	10.4%
**Marital status**		
Single	309	68.1%
Married	140	30.8%
Widowed	3	0.7%
Divorced	2	0.4%
**Kind of device mostly used on social media**		
Laptop	22	5.2%
Cellular	394	92.9%
PC	3	0.7%
Tablet	5	1.2%
**Smoking**		
Yes	89	19.4%
No	370	80.6%
	**Mean**	**SD**
**Age (in years)**	27.29	11.46
**Number of kids**	0.76	1.55
**Number of hours spent on social media per day**	6.22	4.92

### Minimal sample size calculation

According to the G-power software, and based on an effect size f2 = 2%, an alpha error of 5%, a power of 80%, and taking into consideration 14 factors to be entered in the multivariable analysis, and taking a 15% as a refusal rate the results showed that a minimal number of 465 was needed.

### Questionnaire

The questionnaire used during the interview was in Arabic, the native language of Lebanon. The first part assessed the sociodemographic characteristics of the participants (age, number of kids, gender, education level, socioeconomic level and marital status). The second part of the questionnaire consisted of measures used in this study as follows:

### The memory performance scale of the memory awareness rating scale (MARS-MPS)

The MARS-MPS scale was used to assesses views of memory performance on specific aspects of memory functioning following direct experience in each case of an analogue task assessing the given aspect of memory functioning. These tasks are similar to the tasks used in the sub-tests of the Rivermead Behavioural Memory Test Examples of the situations include remembering a person’s name, remembering a short route and recognizing familiar objects. Ratings are made on a 0–4 scale where 0 = very poor and 4 = very good. The maximum possible score is 52 and a higher score indicates a more positive perception of functioning [32]. In this study, the Cronbach alpha for MPS was 0.912.

### Social media use disorder scale (SMUD)

The SMDS is a 27-item scale that measure the degree of problematic social media use [33]. Higher scores indicated higher problematic social media use. The Cronbach’s alpha for this scale was very good (0.847).

### Hamilton depression rating scale (HDRS)

The validated Arabic version of the HDRS was used in this study [34]. The first 17 items of the HDRS are scored and measure the severity of depressive symptoms [35]. The total depression score was calculated by summing the answers of these seventeen items. Higher scores indicated higher depression. The Cronbach’s alpha for this scale was good (0.873).

### Hamilton anxiety scale (HAM-A)

The HAM-A [36], validated in Lebanon [37], is a commonly used scale to measure anxiety in medical and research sites. It consists of 14 items, rated according to a four-point Likert scale (0 = symptoms not present to 4 = very severe symptoms). Higher scores indicated higher anxiety. The Cronbach’s alpha for this scale was good (0.914).

### Beirut distress scale (BDS-10)

It is a 10-item classic stress assessment instrument [38]. The questions in this scale ask about your feelings and thoughts during the last month, with the answers measured on a 4-point Likert scale: 0 (never) up to 3 (very often). Higher scores indicated higher perceived stress. The Cronbach’s alpha for this scale was good (0.743).

### Lebanese insomnia scale (LIS-18)

This 18-item scale is used for the diagnosis of insomnia on the basis of several validated/universally applicable self-report scales [39]. Answers are graded on a 5-point Likert scale (1 = Never to 5 = Always), and items 4, 18 and 22 reversed, with higher scores indicating higher insomnia. The Cronbach’s alpha for this scale was good (0.815).

### Translation procedure of the questionnaire

A forward and backward translation was conducted for the MARS-MPS and PSMU scales. One translator was in charge of translating the scales from English to Arabic, and another one was involved in the translation from Arabic back to English. Discrepancies between the original and translated English versions were resolved by consensus.

### Statistical analysis

SPSS software version 25 was used to conduct data analysis. Cronbach’s alpha values were recorded for reliability analysis for all the scales. The first sample (*n* = 270) was used to conduct the MPS and PSMU items’ factor analysis, whereas the second was used for the confirmatory analysis. A factor analysis was initiated using the “principal component analysis” technique to confirm the legitimacy of the construct of both scales in our sample. The Kaiser-Meyer-Olkin (KMO) value and the Bartlett’s sphericity test were checked for sampling adequacy. The factors with Eigen values > 1 were kept. The Statistica software was used to conduct confirmatory factor analysis on subsample 2 (*n* = 196). Multiple indices of goodness-of-fit were described for each scale: the Relative Chi-square (χ2/df) (cut-off values:< 2–5), the Root Mean Square Error of Approximation (RMSEA) (close and acceptable fit are considered for values < 0.05 and < 0.11 respectively), the Goodness of Fit Index (GFI) and the Adjusted Goodness of Fit Index (AGFI) (acceptable values are ≥0.90) [40].

The Student t-test was used to compare continuous variables in two groups. Pearson correlation was used for linear correlation between continuous variables. The Student t-test was used to compare the means of 2 groups. A stepwise linear regression was conducted, taking the memory performance scale as the dependent variable. All variables that showed a *p* < 0.1 in the bivariate analysis were considered as important variables to be entered in the model in order to eliminate potentially confounding factors as much as possible. Structural equation modeling (SEM) was performed (using SPSS AMOS) to assess the structural association between problematic social media use, stress, insomnia, anxiety, depression and memory performance. The goodness of fit of the model was verified. *P* < 0.05 was considered significant.

### Mediation analysis

The PROCESS SPSS Macro version 3.4, model four [41] was used to calculate three pathways. Pathway A determined the regression coefficient for the effect of problematic social media use on depression/anxiety/stress/insomnia; Pathway B examined the association between depression/anxiety/stress/insomnia and memory performance, independent of the problematic social media use, and Pathway C′ estimated the total and direct effect of problematic social media use and memory performance respectively. Pathway AB calculated the indirect intervention effects. To test the significance of the indirect effect, the macro generated bias-corrected bootstrapped 95% confidence intervals (CI) [41]. A significant mediation was determined if the CI around the indirect effect did not include zero [41]. The covariates that were included in the mediation model were those that showed significant associations with memory performance in the bivariate analysis.

## Results

The mean memory performance score was 31.71 ± 9.88 (median = 31; minimum = 6; maximum = 52), problematic social media use (PSMU) scale 8.15 ± 5.71, depression 9.84 ± 9.14, anxiety 15.00 ± 10.73, stress 18.24 ± 5.28 and insomnia 68.99 ± 12.79.

### Factor analysis of the PSMU and MPS scales

Since the PSMU and MPS scales are not validated in Lebanon, an exploratory factor analysis (EFA) using the principal component analysis was conducted for both scales. A confirmatory factor analysis followed the EFA. Results are summarized in Supplementary Tables 1 and 2. The SMUD and MPS items converged over a solution of 3 and 2 factors respectively.

### Bivariate analysis

Bivariate analysis taking the memory performance scale as the dependent variable showed a negative weakly correlation between number of hours spent on social media per day (*r* = − 0.16, *p* = 0.001), depression (*r* = − 0.16, *p* = 0.001), perceived stress (*r* = − 0.14, *p* = 0.003), anxiety (*r* = − 0.28, *p* < 0.001), problematic Social media use (*r* = − 0.15, *p* = 0.001), insomnia(*r* = − 0.17, *p* < 0.001) and memory performance scale (Table 2).

**Table 2:** Bivariate analysis taking the memory performance scale as the dependent variable

	Memory performance scale	***p***-value
	Mean ± SD	
**Gender**		
Male	30.66 ± 9.34	0.095
Female	32.24 ± 10.07	
	**Correlation coefficient**	
Age	0.156	**0.001**
Number of kids	0.086	0.071
Number of hours spent on social media per day	−0.163	**0.001**
Depression (HAMD scale)	−0.160	**0.001**
Perceived stress (PSS scale)	−0.141	**0.003**
Anxiety (HAMA scale)	− 0.289	**< 0.001**
Problematic social media use	−0.153	**0.001**
Insomnia (LIS scale)	−0.175	**< 0.001**

Numbers in bold indicate significant *p*-values all other variables not displayed in the table showed a *p* > 0.1 in the bivariate analysis and were not included

### Multivariable analysis

The results of a stepwise linear regression, taking the memory performance scale as the dependent variable, showed that higher problematic social media use score (Beta = − 0.21, *p* = 0.008) and higher anxiety (Beta = − 0.25, *p* < 0.001) were significantly associated with lower memory performance scale (Table 3).

**Table 3:** Multivariable analysis: Linear regression taking the memory performance scale variable as the dependent variable

	Unstandardized Beta	Standardized Beta	***p***-value	95% Confidence Interval	
				Lower bound	Upper bound
Anxiety (HAMA scale)	−0.254	−0.275	< 0.001	− 0.338	− 0.169
Problematic social media use score	−0.216	− 0.124	0.008	− 0.376	− 0.056
Perceived stress scale	− 0.102	− 0.055	0.267	− 0.282	0.078
Insomnia	−0.049	− 0.062	0.254	− 0.132	0.035
Depression scale	0.038	0.035	0.528	−0.080	0.157

Variables entered in the model: Problematic social media use score, anxiety (HAMA scale), perceived stress (PSS scale), insomnia (LIS scale) and depression (HAMD scale), gender, age, number of kids.

### Mediation analysis

Higher problematic social media use was significantly associated with higher anxiety, b (coefficient Beta) = 0.2025, 95% BCa CI [0.046, 0.4635], t (the ratio of the difference between the sample mean and the given number to the standard error of the mean) = 2.3475, *p* = 0.0106 (df = 356, R2 = 0.3702). Higher problematic social media use was significantly not associated with lower memory performance even with anxiety in the model, b = − 0.2215, 95% BCa CI [− 0.3834, 0.02–0.05], t = − 2.641.67, *p* = 0.0908; higher anxiety was also significantly associated with lower memory performance, b = − 0.2322, 95% BCa CI [− 0.3331, − 0.1214], t = − 4.255.03, *p* < 0.001 (df = 357, R2 = 0.11). When anxiety was not in the model, higher problematic social media use was significantly associated with lower memory performance, b = − 0.2621, 95% BCa CI [− 0.4240, − 0.1002], t = − 3.162.24, *p* = 0.001025 (R2 = 0.0705, df = 358). The mediating effect of anxiety was 21.19% (partial mediation). Also, a mediation effect of the insomnia was found at 10.52%.

**Fig. 2:**
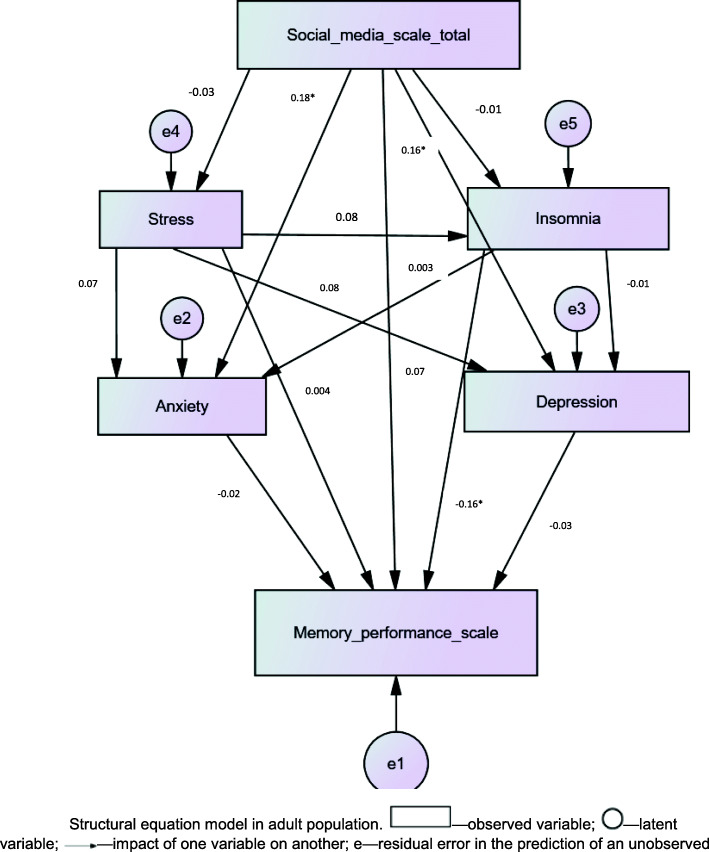
Structural equation model in adult population. —observed variable; —latent variable; —impact of one variable on another; e—residual error in the prediction of an unobserved factor; * *p* < 0.001. Numbers without a * indicate non-significant associations. The values of standardized coefficients are presented

It is noteworthy that depression and, stress or insomnia did not mediate the association between problematic social media use and memory performance (Table 4, Fig. 2).

**Table 4:** Mediation analysis

	**Effect of problematic social media use on anxiety**			**Effect of problematic social media use and anxiety on memory performance**			**Direct effect of problematic social media use on memory performance**			**Mediating effect of anxiety**
	**Beta**	**t**	**p**	**Beta**	**t**	**p**	**Beta**	**t**	**p**	
Problematic social media use	0.25 [0.04–0.46]	2.34	0.01	**−0.15 [−0.34–0.02]**	**−1.67**	**0.09**	**−0.21 [− 0.40—0.02]**	**−2.24**	**0.025**	21.19%
Anxiety				−0.22 [− 0.31—0.14]	−5.03	< 0.001				
	**Effect of problematic social media use on depression**			**Effect of problematic social media use and depression on memory performance**			**Direct effect of problematic social media use on memory performance**			**Mediating effect of depression**
	**Beta**	**t**	**p**	**Beta**	**t**	**p**	**Beta**	**t**	**p**	
Problematic social media use	0.11 [−0.06–0.29]	1.30	0.19	− 0.25 [− 0.44—0.05]	−2.50	**0.01**	−0.26 [− 0.45—0.06]	− 2.62	**0.009**	–
Depression				−0.10 [− 0.21–0.01]	−1.75	0.08				
	**Effect of problematic social media use on stress**			**Effect of problematic social media use and stress on memory performance**			**Direct effect of problematic social media use on memory performance**			**Mediating effect of Stress**
	**Beta**	**t**	**p**	**Beta**	**t**	**p**	**Beta**	**t**	**p**	
Problematic social media use	0.12 [0.01–0.22]	2.25	0.02	−0.18 [−0.37—0.004]	−2.50	0.055	−0.21 [− 0.40—0.02]	−2.24	**0.025**	–
Stress				−0.26 [− 0.44—0.07]	−2.75	**0.006**				
	**Effect of problematic social media use on insomnia**			**Effect of problematic social media use and insomnia on memory performance**			**Direct effect of problematic social media use on memory performance**			**Mediating effect of Insomnia**
	**Beta**	**t**	**p**	**Beta**	**t**	**p**	**Beta**	**t**	**p**	
Problematic social media use	0.24 [−0.008–0.49]	1.89	0.058	−0.19 [− 0.38—0.004]	−2.01	**0.04**	−0.22 [− 0.41—0.03]	−2.28	**0.023**	10.52%
Insomnia				−0.11 [− 0.19—0.03]	−2.80	**0.005**

### Structural equation modeling

The structural relationships between PSMU, insomnia, stress, anxiety, depression and memory performance are displayed in Fig. 2. The path coefficients for the paths from insomnia to memory performance, from PSMU to anxiety and from PSMU to depression were the only ones that showed significance; higher insomnia was significantly associated with less memory performance (SB = -0.16; *p* < 0.001), whereas higher PSMU was significantly associated with higher anxiety (SB = 0.18; *p* < 0.001) and higher depression (SB = 0.16; *p* < 0.001) respectively.

## Discussion

Until this moment, this is the first study conducted among the Lebanese population evaluating the association between problematic social media use and memory work. Our pursuit of this topic is backed up by the fact that recent studies have pointed to a significant up rise in social media activity around the world during the past few years [5, 6, 42]; thus sparking our interest in evaluating the potential deleterious consequences of SNSs (Social Networking Sites). In addition, this study also explores the correspondence between various mood disorders such as depression, anxiety, stress, and insomnia on memory performance. After computing all the data collected, our results clearly stated that the amount of time spent on social media was correlated to lower memory performance as well as increased anxiety, but was not associated with depression and stress.

Our study focused on the effects of problematic social media use on memory performance. In agreement with other studies, the results revealed a clear correlation coupling higher social network sites (SNS) use to lower memory work [16, 17, 43–46]. Literature displayed a modest amount of articles that interpreted this relationship. De facto, this occurrence was primarily linked to “semantic memory” which is the memory of facts and common knowledge, not gained by personal experiences. The constant access to information via the internet will replace the need for certain types of memory, predominantly, the semantic memory. Furthermore, it was demonstrated that increased handling of all activities on the internet will lead to anatomical changes in the grey matter of the brain’s cortex, therefore influencing not only memory but also impulse control and decision making [17]. In other words, social media consumption, and more specifically PSMU, can alter our body’s biological composition in a way that suits the technology most. Given our headed direction in the tech world and the high probability that SNSs are only going to get more popular in the coming years, these findings could represent the starting point of a long-term evolutionary change in the way our minds are structured and the way they process information.

Moreover, our study demonstrated an essential factor that affected memory as well as played a mediating role between our two main variables. This subject was previously discussed and various contradictory theories were given across the literature. On one hand, our results linked anxiety to decreased memory work and this was in opposite to some of the previously published studies [47, 48]. For instance, some demonstrate that symptoms of anxiety alone (excluding symptoms of depression) did not display any significant detrimental consequences on any aspects of memory functioning, in contrast to other types of mood disorders [47]. This could be interpreted through the “lack of motivation” that other types of mood disorders have. Actually, some even go to say that a moderate level of anxiety may have a beneficial outcome on cognition and memory [47]. On the other hand, in correspondence with our results, some studies emphasized that anxiety correlates with lower memory and thus decreased academic performances [11, 49, 50]. This was justified by the possible influence of different mood disorders including anxiety on the verbal working memory and the central executive memory which both are mandatory in educational performance [51]. In fact, worrying takes up a portion of our working memory capacity which means that less residual working memory capacity will be available whenever we are thinking of worrisome affairs [50]. Moreover, a characteristic aspect of anxiety is narrow control on worrying ideas and attentional biases, which can lead to greater focus on negative stimuli disrupting memory and cognitive performances [52]. Furthermore, the relationship between anxiety and addictive disorders is a bidirectional relationship. People suffering from anxiety are more at risk of developing addictive disorders such as PSMU [23], and PSMU leads to increased anxiety due to the famous “fear of missing out” [53]. Overall, all this information could also explain anxiety’s partial mediation role between PSMU and memory performance. Provided the divergence in results across the literature, and considering that we cannot ignore the potential threat of anxiety influencing our youth’s memory and cognitive functions, we find it ever so important to encourage deeper investigations concerning this topic with the purpose of unraveling the truth behind these interactions. For that, we believe our findings will play a role in clearing and detailing the extremely heterogeneous image of anxiety disorders and their correlations with cognitive deviations, all whilst refining our diagnostic skills.

Finally, multivariable analysis results did not associate depression, stress and insomnia to lower memory performance. These findings oppose previous literature [47, 50, 51]. In fact, it has been suggested that depressive symptoms alone (excluding symptoms of anxiety) affect the prompt recall of new information and their amount of acquisition, but did not affect either retrieval nor retention of memory [47]. The latter study suggested that memory difficulties are mostly reported in individuals who suffers from coexistent mood disorders [47]. However, our results did not demonstrate such relationship. This can be explained by the fact that the depression, anxiety, stress and insomnia scales were all self-reported questionnaires and were not administered by a psychologist or psychiatrist, therefore possibly affecting said results’ reliability. Similarly, the memory awareness rating scale (MARS) was not carried out by specialists, which might explain the negative association concluded.

### Clinical implications

If correlation can be made through this study in Lebanon, awareness about social media should be spread among parents and children in order to reduce its use and prioritize activities that promote teenagers executive functioning and well-being such as sleep, physical activity along with positive interactions with family and friends. In addition, this study can open the gate for future research that should focus on prevention programs and possible interventions to mitigate the potential adverse consequences among those that suffer from problematic SNSs overuse. Plus, if an individual is already known to have a PSMU, it would be of a great importance to look for signs and symptoms of other comorbid mood disorders such as anxiety in order to treat it and control it in early stages and prevent further damage. Finally, in Lebanon, the proportion of people being treated for mental disorders turned out to be much lower than more developed countries [27] and since our study calls attention on the deleterious consequences of anxiety on memory thus affecting academic performance, schools, universities and work places across Lebanon must implement more psychologists and more interventional programs in order to assist and provide adequate treatment to the people in need.

### Limitations

First, this is a cross-sectional study therefore, it cannot assess the causality of relationships. Second, all variables were evaluated through a self-reported questionnaire and not through a clinical interview by a health care professional, thus, the responses might lack precision and accuracy may have been subject to reporting bias. Likewise, information bias may be present on account of problems in question understanding, recall deficiency and over or under evaluating symptoms. Besides, due to the retrospective nature of our study, a recall bias might be possible. A selection bias can also be considered because of the refusal rate for participation and since the sample is represented by more females than males and by persons with a considerably young age. Also, the mean age of the participants was low, with females being more represented numerically than males, thus results cannot be extrapolated to the general population, with potential impact on outcome variables like depressive and anxiety symptoms. Also, a weak correlation was found between variables. Residual confounding bias is also possible, since there could be factors such as such as social media platforms used, interactions in social media, chat or message, or internet access that were not measured in this study. Finally, based on the limitations described above, the findings of this study cannot be generalized to the whole population.

## Conclusion

To conclude, concerning problematic social media use, a clear correlation was demonstrated in this study linking it to lower memory performance, with this association being partially mediated by anxiety. Future studies should evaluate the possible mechanisms and methods for effective awareness especially towards the younger generation. Concerning anxiety, it obviously affected certain types of memory leading to lower memory functioning and thus decreased academic performance. Therefore, it is a must to highlight the importance of diagnosing and treating anxiety and depression that are recently increasing over the decades in order to avoid their negative sequels.

## Supplementary Information

**Additional file 1: Supplementary Table 1.** Factor structured of the social media use disorder scale. SMUD=Social media use disorder. Confirmatory factor analysis for the PSMU scale. A confirmatory factor analysis was run on Sample 2 (*n* = 196), using the three-factor structure obtained in Sample 1. The results were as follows: the Maximum Likelihood Chi-Square = 68.88 and Degrees of Freedom = 25, which gaPSMUDe a χ2/df = 2.75. For non-centrality fit indices, the Steiger-Lind RMSEA was 0.092 [0–0.142], the Joreskog GFI, 0.922, and AGFI 0.901. **Supplementary Table 2.** Factor structured of the memory performance scale. Confirmatory factor analysis of the MPS scale. A confirmatory factor analysis was run on Sample 2 (*n* = 196), using the two-factor structure obtained in Sample 1. The results were as follows: the Maximum Likelihood Chi-Square = 475.34 and Degrees of Freedom = 148.44, which gave a χ2/df = 3.20. For non-centrality fit indices, the Steiger-Lind RMSEA was 0.118 [0.106–0.130], the Joreskog GFI, 0.855, and AGFI 0.880.

## Data Availability

The authors do not have the right to share any data information as per their institutions policies.
