# Supplementary material for: Association between problematic social media use and memory performance in a sample of Lebanese adults: the mediating effect of anxiety, depression, stress and insomnia
Source: Head Face Med. 2021 Feb 23;17:6. doi: 10.1186/s13005-021-00260-8 (PMC7901207; doi:10.1186/s13005-021-00260-8)
Supplement: Supplementary file 1 — Additional file 1: Supplementary Table 1. Factor structured of the social media use disorder scale. SMUD=Social media use disorder. Confirmatory factor analysis for the PSMU scale. A confirmatory factor analysis was run on Sample 2 (n = 196), using the three-factor structure obtained in Sample 1. The results were as follows: the Maximum Likelihood Chi-Square = 68.88 and Degrees of Freedom = 25, which gaPSMUDe a χ2/df = 2.75. For non-centrality fit indices, the Steiger-Lind RMSEA was 0.092 [0–0.142], the Joreskog GFI, 0.922, and AGFI 0.901. Supplementary Table 2. Factor structured of the memory performance scale. Confirmatory factor analysis of the MPS scale. A confirmatory factor analysis was run on Sample 2 (n = 196), using the two-factor structure obtained in Sample 1. The results were as follows: the Maximum Likelihood Chi-Square = 475.34 and Degrees of Freedom = 148.44, which gave a χ2/df = 3.20. For non-centrality fit indices, the Steiger-Lind RMSEA was 0.118 [0.106–0.130], the Joreskog GFI, 0.855, and AGFI 0.880. [file 13005_2021_260_MOESM1_ESM.docx]

**Supplementary file**

| **Supplementary Table 1: Factor structured of the social media use disorder scale.** | | | |
| --- | --- | --- | --- |
| Items | **Factor 1** | **Factor 2** | **Factor 3** |
| SMUD 1 |  |  | 0.459 |
| SMUD 2 | 0.640 |  |  |
| SMUD 3 |  | 0.808 |  |
| SMUD 4 |  |  | 0.632 |
| SMUD 5 |  | 0.534 |  |
| SMUD 6 | 0.524 |  |  |
| SMUD 7 |  |  | 0.452 |
| SMUD 8 | 0.516 |  |  |
| SMUD 9 |  | 0.643 |  |
| SMUD 10 |  |  | 0.562 |
| SMUD 11 |  | 0.326 |  |
| SMUD 12 | 0.470 |  |  |
| SMUD 13 |  | 0.473 |  |
| SMUD 14 |  | 0.528 |  |
| SMUD 15 |  | 0.614 |  |
| SMUD 16 |  |  | 0.702 |
| SMUD 17 |  | 0.691 |  |
| SMUD 18 |  | 0.439 |  |
| SMUD 19 |  |  | 0.792 |
| SMUD 20 | 0.698 |  |  |
| SMUD 21 | 0.437 |  |  |
| SMUD 22 | 0.512 |  |  |
| SMUD 23 |  |  | 0.407 |
| SMUD 24 |  | 0.576 |  |
| SMUD 25 |  |  | 0.717 |
| SMUD 26 | 0.723 |  |  |
| SMUD 27 | 0.360 |  |  |
| **Proportion of variance explained (%)** | 0.306 | 0.065 | 0.057 |

SMUD=Social media use disorder

**Confirmatory factor analysis for the PSMU scale**

A confirmatory factor analysis was run on Sample 2 (n=196), using the three-factor structure obtained in Sample 1. The results were as follows: the Maximum Likelihood Chi-Square = 68.88 and Degrees of Freedom = 25, which gaPSMUDe a χ2/df = 2.75. For non-centrality fit indices, the Steiger-Lind RMSEA was 0.092 [0–0.142], the Joreskog GFI, 0.922, and AGFI 0.901.

| **Supplementary Table 2: Factor structured of the memory performance scale** | | |
| --- | --- | --- |
| **Items** | **Factor 1** | **Factor 2** |
| MPS 1 | .887 |  |
| MPS 2 | .862 |  |
| MPS 5 | .761 |  |
| MPS 4 | .634 |  |
| MPS 7 | .612 |  |
| MPS 6 | .577 |  |
| MPS 3 | .453 |  |
| MPS 10 |  | .851 |
| MPS 11 |  | .835 |
| MPS 12 |  | .814 |
| MPS 13 |  | .654 |
| MPS 8 |  | .619 |
| MPS 9 |  | .545 |
| **Percentage of variance explained (%)** | 48.67 | 7.75 |

**Confirmatory factor analysis of the MPS scale**

A confirmatory factor analysis was run on Sample 2 (n=196), using the two-factor structure obtained in Sample 1. The results were as follows: the Maximum Likelihood Chi-Square = 475.34 and Degrees of Freedom = 148.44, which gave a χ2/df = 3.20. For non-centrality fit indices, the Steiger-Lind RMSEA was 0.118 [0.106–0.130], the Joreskog GFI, 0.855, and AGFI 0.880
